# Association between stress and bilateral symmetrical alopecia in free-ranging Formosan macaques in Mt. Longevity, Taiwan

**DOI:** 10.1038/s41598-021-90725-2

**Published:** 2021-05-27

**Authors:** Chen-Chih Chen, Ai-Mei Chang, Ming-Shan Tsai, Yen-Hua Huang, Kurtis Jai-Chyi Pei, Yi-Chia Li

**Affiliations:** 1 Institute of Wildlife Conservation, College of Veterinary Medicine, National Pingtung University of Science and Technology, Pingtung, 912 Taiwan, ROC; 2 International Program in Animal Vaccine Technology, International College, National Pingtung University of Science and Technology, Pingtung, 912 Taiwan, ROC; 3 Wildlife Conservation Research Unit, Department of Zoology, University of Oxford, Oxfordshire, OX13 5QL UK; 4 Department of Forest and Wildlife Ecology, University of Wisconsin-Madison, Madison, WI 53706 USA; 5 Department of Veterinary Medicine, College of Veterinary Medicine, National Pingtung University of Science and Technology, Pingtung, 912 Taiwan, ROC; 6 Research Center for Animal Biologics, National Pingtung University of Science and Technology, Pingtung, 912 Taiwan, ROC

**Keywords:** Zoology, Ecology, Endocrinology

## Abstract

Since 2013, a high incidence of bilateral symmetrical alopecia has been observed in free-ranging Formosan macaques (*Macaca cyclopis*) in Mt. Longevity, Taiwan. We hypothesized that stress induces alopecia in this population. To verify our hypothesis, we evaluated the histopathological characteristics of skin biopsy and used a validated enzyme immunoassay (EIA) for fecal glucocorticoid metabolite (FGM) analysis, which act as an indicator of stress experienced by the individual. Follicular densities were lower (2.1–3.0 mm^2^) in individuals with symmetrical alopecia than in those with normal hair conditions (4.7 mm^2^). Furthermore, anagen to catagen/telogen ratios were lower in individuals with alopecia (0–1.4) than in those with normal hair (4.0). The histopathological characteristics of alopecia were similar to those of telogen effluvium, which indicates that stress is one of the possible etiologies. On the basis of the analytical and biological validation of EIAs for FGM analysis, 11β-hydroxyetiocholanolone was considered suitable for monitoring adrenocortical activity in both sexes of Formosan macaques. The mean concentrations (standard error; sample size) of 11β-hydroxyetiocholanolone were 2.02 (0.17; n = 10) and 1.41 (0.10; n = 31) μg/g for individuals with and without alopecia, respectively. Furthermore, the results of logistic regression analysis show that 11β-hydroxyetiocholanolone (*p* = 0.012) concentration was positively associated with alopecia. Thus, stress was the most likely to trigger symmetrical alopecia in Formosan macaques in Mt. Longevity. Although stress can decrease the fitness of an individual, considering the population status of Formosan macaques in Taiwan is stable and alopecia was only observed in our study area, which is isolated from other populations, the impact on the total population of Formosan macaque in Taiwan is limited. Nonetheless, stress-induced immunosuppression and alopecia might affect the local abundance and increase zoonosis risk due to frequent human–macaque contact in Mt. Longevity. Future studies are suggested to focus on the causative factor of stress and the effects of stress and alopecia on the health and welfare in the Formosan macaques.

## Introduction

**Figure 1:**
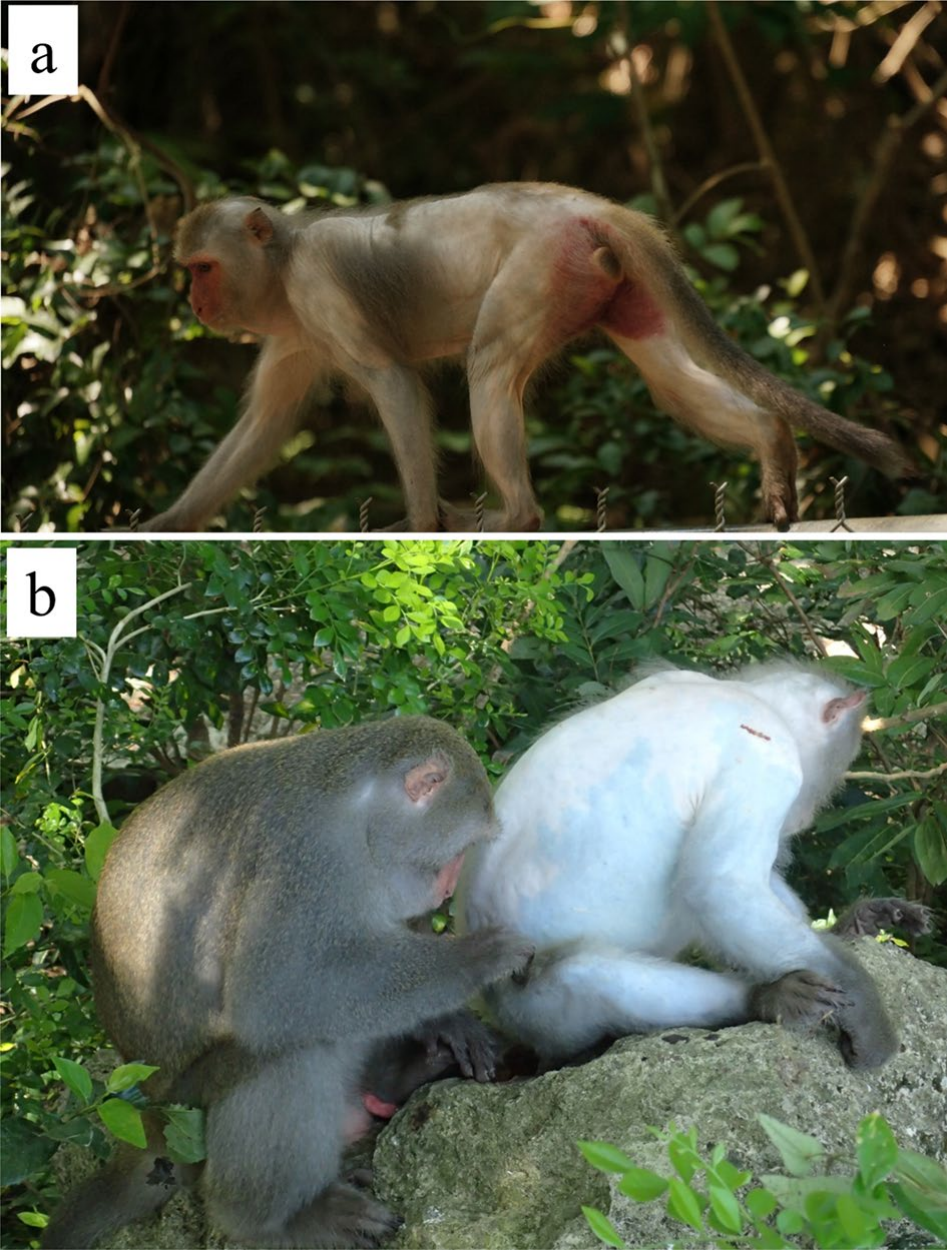
(**a**) An adult female Formosan macaque. (**b**) An adult male Formosan macaque on the right side with severe bilateral symmetrical alopecia compared with the Formosan macaque on the left side with normal hair.

**Figure 2:**
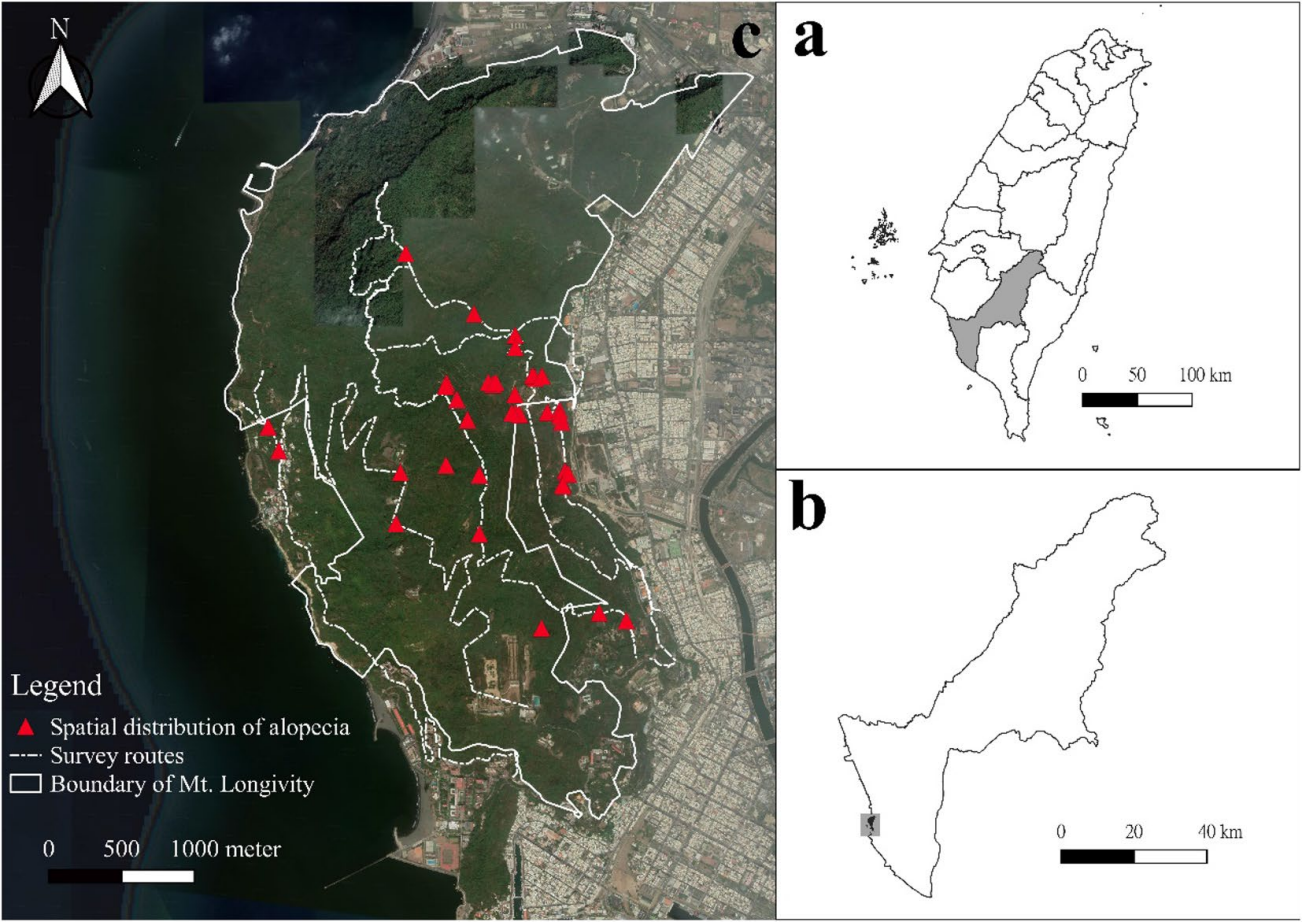
Study area, and the distribution of Formosan macaques with bilateral symmetrical alopecia in Mt. Longevity, SNNP, Taiwan. (**a**) Gray area indicates Kaohsiung city in Taiwan. (**b**) Mt. Longevity (gray area) is located on the west side of Kaohsiung city. (**c**) Map of Mt. Longevity, and the spatial distribution of individuals with bilateral symmetrical alopecia.

Since 2013, a high incidence of bilateral symmetrical alopecia was observed in free-ranging Formosan macaques (*Macaca cyclopis*) (Fig. 1) in Mt. Longevity (Fig. 2), Shoushan National Nature Park (SNNP), Taiwan. However, the cause of alopecia was unknown. The bilateral symmetrical alopecia has been described in different macaque species in captivity^1–3^, however, based on our knowledge, it has never been described in free-ranging macaques.

Alopecia is a medical term for hair loss, which could be occurred in part or completely on the body with normal hair present. Animal hair functions as an anatomical and physiological barrier for the animal against environmental hazards^4^. The occurrence of alopecia in an individual of wildlife may affect the stability of thermoregulation and increase the energy expenditure to maintain a suitable body temperature^5^. Overall, alopecia can influence the body condition, survival, and fitness of wildlife^5–7^. Furthermore, Alopecia or hair loss can be an indicator of health problems. Alopecia is commonly noted in captive macaques^8^. Factors that contribute to alopecia onset in nonhuman primates have been categorized as natural causes, such as aging, and seasonal changes, psychological factors^9^, nutritional deficiency^10^, hormonal imbalance^11^, immunological diseases^12^, and allergic reactions^13^. However, psychological factors were considered to lead to alopecia through the stimulation of abnormal hair-plucking behavior in macaques^14^. Recent studies have indicated that psychological stress is a direct cause of alopecia and is positively correlated with alopecia severity in rhesus monkeys^2, 8^. Studies on mice have indicated that stress can induce telogen effluvium leading to alopecia through the possible mechanism of production of catagen-inducing hair growth inhibitors and hair-damaging proinflammatory cytokines^15^.

Prolonged exposure of animals to stressors results in a high concentration of GC secretion, which has been shown to result in decreased individual survival and fitness through immunosuppression, muscle wasting, and reduced reproduction^16–18^. Therefore, GCs are commonly used to monitor chronic stress and identify possible stressors in both captive and free-ranging wild animals^19^.

After GCs are secreted from the adrenal cortex into the blood, they are metabolized by the liver and kidney and eliminated from the animal’s body through urine or feces^20^. For measuring the amount of GCs secreted, collecting noninvasive samples, such as feces^21^, is recommended and is commonly adopted. Comparing to blood samples, collecting feces avoids confounding increase of GCs secretion during the restriction period^22^. In addition, fecal samples can be collected repeatedly and easily, without interfering behavior and endocrine status of an animal, which allows for the long-term and repeated monitoring of changes in GC concentrations in response to a specific stimulus. Furthermore, GC metabolites accumulate in feces over a certain period, and their concentrations are less affected by periodic fluctuations or the pulsatility of GC secretion^22, 23^. This property is particularly advantageous for studying wild animals^21, 24^.

Although using fecal glucocorticoid metabolite (FGM) concentration to represent the stress status of an animal has its advantages, problems related to FGM analysis must be considered before the analysis. GCs are metabolized before elimination from an animal’s body, and therefore, little or no native GCs may remain in feces or urine^25^. Furthermore, the primary route of the excretion of GC metabolites through urine or feces considerably varied among species^26, 27^. Moreover, the metabolic process of GCs can substantially vary between species and sexes and even between phylogenetically closely related species, resulting in numerous different metabolites^23, 25, 28^. Overall, validating all the procedures of FGM analysis for each specific species and sex is essential to accurately measuring FGMs and determining the stress status of individuals^20, 23, 27, 29^. Usually, hormone concentrations are identified using immunoassays with hormone- or hormone-group-specific antibodies to bind with the target hormone or hormone metabolites. Because native GCs are negligible or nonexistent in feces, selecting appropriate antibodies based on analytical and physiological (or biological) validation is crucial to obtaining meaningful results^19, 30, 31^.

The stress-induced alopecia in Formosan macaques was suspected. We performed a skin biopsy of individuals with symmetrical alopecia for histopathological assessment to determine the possible etiology. In addition, to clarify the association between alopecia and GC secretion, we first evaluated the validity of four enzyme immunoassays (EIAs) for monitoring adrenocortical activity through the quantification of FGMs in Formosan macaques. Furthermore, we used the validated EIAs to determine FGM concentrations of fecal samples collected from individuals with and without alopecia. We hypothesized that the level of GCs excreted was positively associated with bilateral symmetrical alopecia in Formosan macaques.

## Materials and methods

### Ethics statement

The permission for trapping Formosan macaques in Mt. Longevity was obtained from the Forest Bureau (Permit no.: COA, Forestry Bureau, 1041701001). The procedures for trapping Formosan macaques in Mt. Longevity, administering anesthesia, and collecting samples were approved by Institutional Animal Care and Use Committee (IACUC) of National Pingtung University of Science and Technology (Approval no.: NPUST-104-021). All the procedures that involve live animals were performed in compliance with the IACUC regulations and Animal Research: Reporting of In Vivo Experiments (ARRIVE) guidelines. The fecal sample collection in Pingtung Rescue Center for Endangered Wild Animals (PTRC) was approved by PTRC (Approval no.: PTRC-104061501). No other permit was needed for collecting fecal samples from macaques in Mt. Longevity. All details on sampling methods are provided in the following sections.

### Study area

This study was conducted in Mt. Longevity, which is located in SNNP (latitude: 22.650 and longitude: 120.260) on the west side of Kaohsiung city (Fig. 2). A city and an ocean isolate Mt. Longevity from other natural habitats. The terrain of Mt. Longevity is characterized by uplifted coral reefs and limestone covered with natural forest. Formosan macaques are endemic to Mt. Longevity. The estimated population size of Formosan macaques ranged from 1200 to 1500 in the sampling area from 2012 to 2015^32^. The density of Formosan macaques in Mt. Longevity was high for a long period owing to food provision for a long term history^33^. The average density of Formosan macaques on Taiwan island was 0.72 troops/km^34^; however, its population density in Mt. Longevity was 5.72 troops/km^35^. Food provision to macaques is prohibited, and a law was strongly enforced against food provision after the establishment of SNNP in 2011, which may have limited the food for macaques in Mt. Longevity.

### Histopathological evaluation of alopecia

Free-ranging Formosan macaques with symmetrical alopecia were trapped for the histopathological evaluation of skin tissues with alopecia. We used steel-mesh box traps (Tomahawk Live Trap, LLC., Hazelhurst, WI, USA) modified for remote access, and a banana was used as a bait. Individuals with severe symmetrical alopecia were trapped. We adopted the alopecia severity scoring system for primates developed at the Washington National Primate Research Center^36^. Briefly, the scoring system separates the body into 12 parts and estimates the percentage of the body affected by alopecia. The alopecia score ranges from 0 to 4, indicated from no body part affected to more than 9 body parts affected by alopecia, respectively^36^. We excluded the non-symmetrical alopecia from data analysis, which possibly due to wound scar. We define that the score of 4 as severe alopecia. The trapped macaques were anesthetized by veterinarians with a mixture of dexmedetomidine hydrochloride (25 µg/kg) and tiletamine HCl/zolazepam HCl (2 mg/kg). Skin tissues of the back area with alopecia of approximately 1 cm^2^ were surgically collected and fixed in 10% neutral buffered formalin for 24 h and embedded in a single paraffin wax block for histological evaluation. After recovery from anesthesia, the trapped macaques were released and continuously tracked in the following days to ensure the healing of the biopsy wound. Vertical and transversal sections (5 μm) at the reticular dermis layer of skin biopsy were mounted onto a glass slide and dried by heating at 37 °C for 24–36 h. The slide section was deparaffinized and rehydrated through a graded series of ethanol baths and stained using hematoxylin and eosin stain. The slides were left to dry in an incubator at 37 °C for 4–8 h and mounted with cover slip. We examined the histopathological changes in skin sections to evaluate the possible etiology of alopecia. In addition, transversal skin sections were used for obtaining follicular counts and determining follicular stages, namely anagen, catagen, and telogen, based on Whiting^37^. We estimated follicular density per square millimeter by dividing the total count of follicles by the area covered in the image by using ImageJ software^38^. The follicles on the edge of the image were not counted. Moreover, we grouped catagen and telogen follicles together and estimated the anagen to catagen/telogen ratio^39^.

### EIA validation

#### Stress manipulation of Formosan macaques in captivity

We adopted a biological validation procedure to induce GC secretion through restraint and anesthesia procedures^30, 40, 41^ for routine health evaluation in captivity. We then assessed whether the increased GC secretion could be detected based on different EIA FGM quantities. In addition, we evaluated the capacity and specificity of different antibodies to quantify FGM abundance through integration with high-performance liquid chromatography (HPLC) analysis. Lastly, we compiled the results of the EIA and HPLC to evaluate the suitability of four EIAs in assessing adrenocortical activity in Formosan macaques.

For validating the EIAs, fresh fecal samples were collected for 5 consecutive days from one male and one female captive adult Formosan macaque housed individually in the Pingtung Rescue Center for Endangered Wild Animals (PTRC), National Pingtung University of Science and Technology. The individual cages used for housing the macaques were 2 m high and 1 m wide and deep; the upper area of the cage had a platform for resting.

Pooled fecal samples of 1 day of each individual were collected by the PTRC staff before and after an anesthesia procedure for routine health evaluation of each individual. Anesthesia was administered to stimulate stress (physical challenge) in the macaques on the third day of sample collection. Collected feces were stored at − 20 °C immediately until analysis. The feces collected before the health check were used for baseline analysis, and the samples collected after the health check were used for evaluating the adrenal responses to stress.

#### Extraction of fecal hormone metabolites

Collected feces were transferred to 15-mL centrifuge tubes and covered with parafilm. Holes were made on the parafilm to allow evaporation. Lyophilization was then performed for 72 h. Dehydrated feces were filtered to exclude large particles, and the rest were ground to powder. The fecal powder of 50 mg was transferred to new 15-mL centrifuge tubes, and 3 mL of 80% methanol was then added. After vortexing for 10 min and centrifuging at 3000 rpm for 10 min, the supernatant was transferred to a 5-mL vial, labeled, and stored at − 20 °C until further analysis.

### HPLC analysis

The samples collected at the PTRC from one male and one female macaque were used for reverse-phased high-performance liquid chromatography. To purify the fecal extractions, 1 mL of the fecal extract was mixed with 3 mL of sodium acetate buffer (0.2 M, pH 4.2) and filtered using solid-phase extraction (SPE) cartridges (Sep-pak C18 cartridge 1 g, Waters, MA, USA). The SPE cartridge was activated by pumping 10 mL of 100% methanol and 10 mL of double-distilled water slowly with a syringe before addition of the extraction mixture. After the total volume of the extraction mixture was passed, 10 mL of double-distilled water was pumped using a syringe to wash the cartridge. The elution was discarded, and the cartridge was left to dry at room temperature for 3 h. After drying, 10 mL of methanol (100%) was added to elute the extraction from the cartridge. The elution was collected and evaporated to dryness through nitrogen sweeping and then reconstituted in 400 μL of 40% Acetonitrile (ACN) buffer for the HPLC process to separate the steroids. The HPLC settings include a flow speed of 0.4 mL/min and ACN:H_2_O ratio of 40:60 as mobile phase before injection of 100 μL of the purified extraction. Fractions (n = 80, 400 μL each) were collected every minute and were evaporated to dryness. An assay buffer (1 mL) was then added to each fraction and stored at − 20 °C before the EIA analysis. Moreover, the standards of the four selected FGMs (cortisol, corticosterone, 11-oxoaetiocholanolone, and 11β-hydroxyetiocholanolone) were separated into fractions with HPLC to obtain elution positions for reference.

### Enzyme immunoassays

Four competing EIAs, namely cortisol, corticosterone, 11-oxoaetiocholanolone, and 11β-hydroxyetiocholanolone, were used to detect targeted steroids and metabolites of HPLC elution and fecal extractions, as described by Heistermann et al.^30^. The antibody, standard, and biotin-labeled steroid of each target used in this study were kindly provided by Dr. Palme from the Department of Biomedical Sciences, University of Veterinary Medicine, Vienna, Austria. The cross-reactivities of all antibodies applied in this study were described by Heistermann et al.^30^. The EIAs were performed using 96-well microtiter plates (MTPs) coated with protein A (P-7837, Sigma-Aldrich, Vienna, Austria), according to procedures described by Palme and Möstl^42^. In brief, samples were diluted 20 times with an EIA assay buffer before reaction. Biotin-labeled steroids (100 μL) and diluted samples or standards (50 μL), as well as antibodies (100 μL), were added to each well and incubated overnight at 4 °C. An enzyme solution (250 μL/well) containing streptavidin–horseradish peroxidase conjugate (RPN1231, GE Healthcare) was added to the plate after four washes with 0.02% Tween 20 (VE-V900548, Vetec) and then incubated with mild shaking in the dark at 4 °C for 45 min. The plate was washed again before the addition of 250 μL of substrate solution containing tetramethylbenzidine (SI-T2885, Sigma-Aldrich) and incubated with mild shaking in the dark for another 45 min at 4 °C. After incubation, 50 μL of 10% H_2_SO_4_ was added to each well to stop the reaction, and the plate was read using a multimode microplate reader (Corona Electric Co., Ltd, Japan) within 30 min to measure absorbance at 450 and 620 nm as measuring and reference filters, respectively. The differences in both readings were used for obtaining the standard curve formula, which was then used for the concentration calculation of each sample. Intra- and interassay coefficients of variance (CVs) of low and high concentration pool samples were calculated for quality control in EIA analysis. Only MTPs with less than 15% CV were considered valid results^43^.

### Fecal sample collection from Formosan macaques in Mt. Longevity and FGMs analysis

To evaluate the correlation between stress and alopecia, we collected fecal samples of Formosan macaques with and without alopecia by following troops with bilateral symmetrical alopecia individuals in Mt. Longevity. The research crew was familiar with the macaque troops and individuals in the troops^35, 44^. During the study period, the individuals with alopecia from different troops were recognized and monitored, which facilitated avoidance of repeating sampling of the same individual. When field crews observed a macaque defecating, a fecal sample was collected and stored in a coolbox immediately. The feces were then stored at − 20 °C. We recorded the age, sex, and status of individuals with alopecia while collecting fecal samples. Age was recorded as juvenile, subadult, and adult according to the classification by Hsu and Lin^45^. We did not collect the sample from infants in this study due to the limitation of availability. The procedures of fecal glucocorticoid metabolites extraction and EIA was the same as the description above.

### Data analysis

We used a multivariate logistic regression analysis to evaluate the relationship between alopecia, stress, and other possible covariates, such as age and sex. The analysis was conducted using the Generalized Liner Model (GLM) module in the computing environment R (R Development Core Team, 2010). The alopecia status during fecal sample collection was treated as the dependent variable. The fecal glucocorticoid metabolites concentrations of 11β-hydroxyetiocholanolone was treated as explanatory variables. Furthermore, age and sex variables were treated as possible confounders for data analysis. We first transformed all the explanatory variables to dummy variables and centered or standardized the numerical variables. Multicollinearity between the explanatory variables was evaluated using the variance inflation factor (VIF)^46^. The VIF threshold was set as 10 to avoid the problematic effect of multicollinearity on parameter estimations^47^. Variables were discarded from model construction if the VIF value was more than 10. The explanatory variables selected in the models were based on the Wald test with a *p* threshold value of 0.05^48, 49^. We compared the model fit based on the Akaike information criterion (AIC), with lower AIC values indicating a better model fit to the dataset.

## Results

### Histopathological evaluation of alopecia

**Figure 3:**
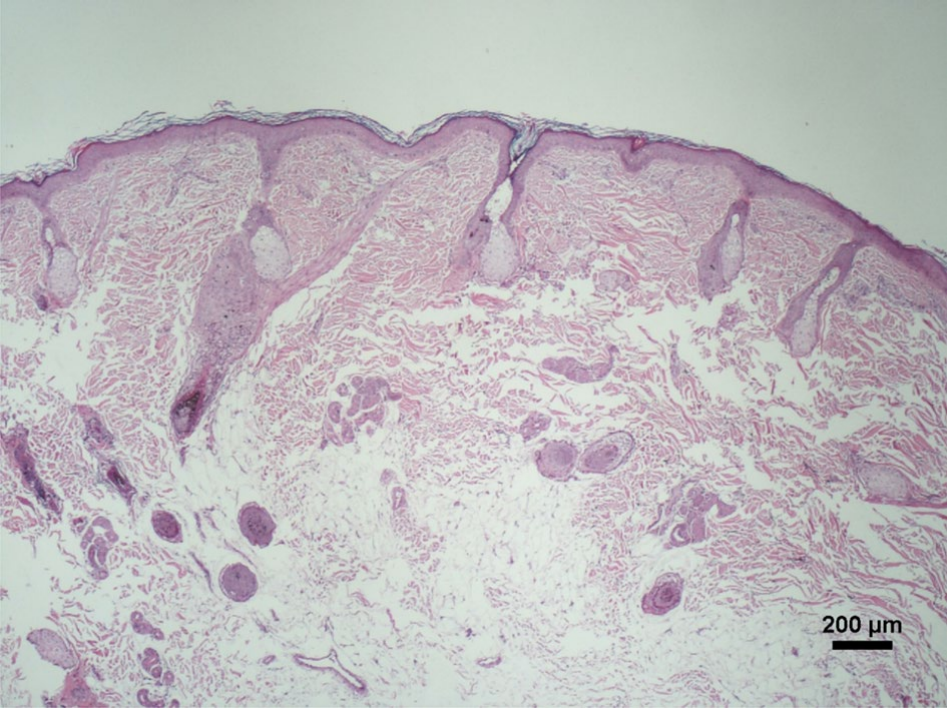
Vertical section of skin biopsy of a macaque (No. 2016080403) with symmetrical alopecia showing no infiltration of inflammatory cells or pathogen infection in skin tissues.

**Figure 4:**
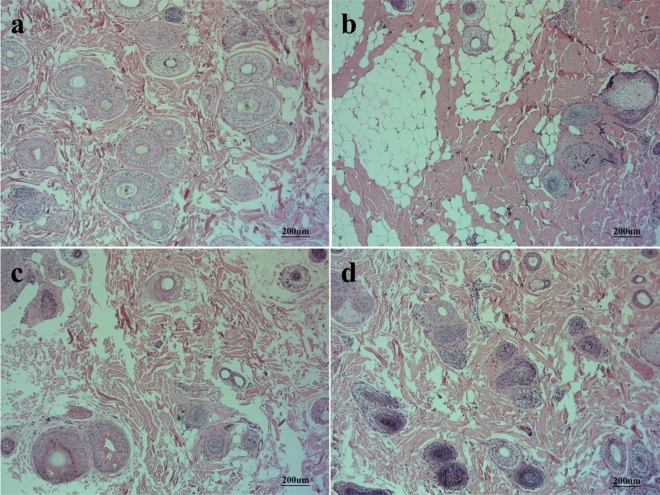
Histopathological characteristics of a transverse skin section at the reticular dermis layer of Formosan macaques with and without symmetrical alopecia. (**a**) C2016091201, individual with normal hair appearance; (**b**) 2016080401; (**c**) 2016080402; and (**d**) 2016080403 were three individuals with symmetrical alopecia. A higher proportion of catagen/telogen was noted in macaques with alopecia than in those with normal hair appearance.

We trapped three Formosan macaques of different ages, sexes, and rankings with severe symmetrical alopecia (Table 1). The skin sample of a roadkilled Formosan macaque in the study area with normal hair appearance was used to compare the histopathological changes in skin samples of symmetrical alopecia Formosan macaques. In the vertical sections of skin tissues of alopecia individuals, we did not observe the infiltration of inflammatory cells of skin tissues and pathogen infection, such as infection with fungi and ectoparasites. Therefore, alopecia due to infection and scarring was ruled out (Fig. 3). The transverse skin sections of individuals with symmetrical alopecia revealed lower follicular densities (1.6–3.9/mm^2^) than in individual with normal hair condition (4.7/mm^2^; Table 2). In addition, anagen to catagen/telogen ratios were lower in alopecia individuals (0–1.4) than in individual with normal hair (4.0). A lower anagen to catagen/telogen ratio indicated a higher proportion of catagen and telogen follicles in individuals with symmetrical alopecia (Table 2; Fig. 4).

**Table 1:** Individual information of Formosan macaques with symmetrical alopecia and a reference individual (no. C2016091201) with normal hair appearance for a comparison of their histopathological results.

Individual no	2016080401	2016080402	2016080403	C2016091201
Appearance				
Alopecia	Yes	Yes	Yes	No
Age	Adult	Adult	Subadult	Adult
Sex	Male	Female	Male	Female
Ranking	High	Low	Medium	Unknown

**Table 2:** Estimation of follicular density, stages, and anagen to catagen/telogen ratio for the skin biopsy of Formosan macaques with symmetrical alopecia in comparison with individuals with normal hair appearance.

Individuals	Area (mm^2^)	Anagen	Catagen/telogen	Vellus	Total follicle	Density (mm^2^)	A/CT ratio^a^
C2016091201	3.8	12	3	3	18	4.7	4.00
2016080401	3.8	3	3	0	6	1.6	1.00
2016080402	3.8	0	6	2	8	2.1	0.00
2016080403	3.8	7	5	3	15	3.9	1.40

^a^A/CT ratio denotes anagen to catagen/telogen ratio.

### Validation of FGM analysis with HPLC and EIA

**Figure 5:**
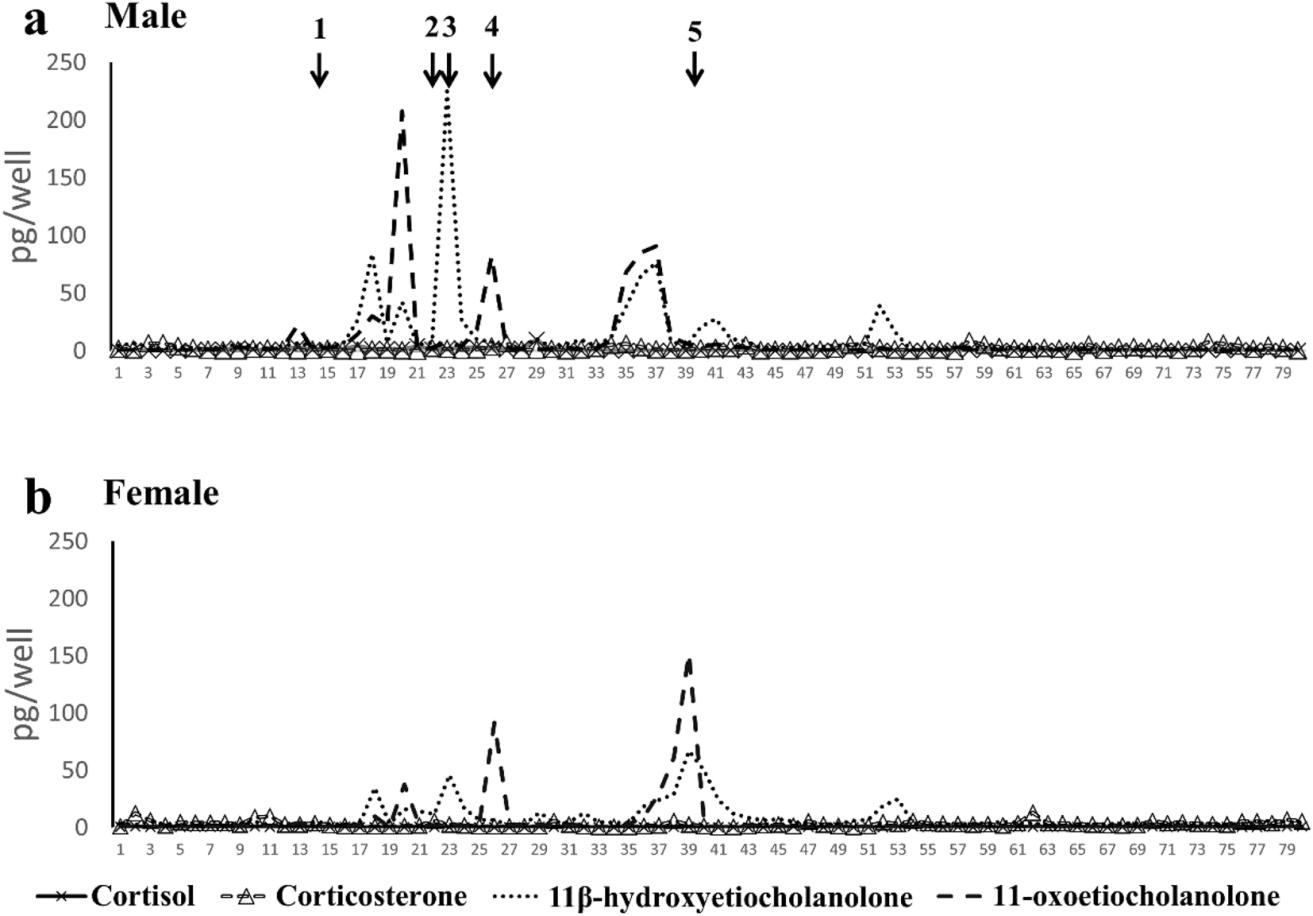
Immunoreactivity of HPLC elutions of Formosan macaque FGMs detected through EIA analyses for cortisol, corticosterone, 11β-hydroxyetiocholanolone, and 11-oxoetiocholanolone following anesthesia stimulation. The number and arrow above HPLC profiles indicate the elution positions of reference standards: (1) cortisol (14); (2) corticosterone (22); (3) 11β-hydroxyetiocholanolone (23); (4) 11-oxoetiocholanolone (26); and (5) testosterone (40).

The results of the four EIA analyses of all HPLC fractions collected from one male and one female are shown in Fig. 5. The 11-oxoetiocholanolone was mainly eluted at positions 18, 23, and 37–38 in both sexes. 11β-Hydroxyetiocholanolone was mainly eluted at positions 20, 26, and 37–39 in both sexes. Both cortisol and corticosterone were presented in low amounts in all fractions, and they neither showed an obvious peak after EIA analysis nor coelution with authentic cortisol at position 14 and with authentic corticosterone at position 22 (Fig. 5). Most of the immunoassay reactions were located before position 40, which has been referred to as elution positions of GC metabolites and different from other steroids with similar structure, such as testosterone ^30, 50, 51^ (Fig. 5).

### FGM baseline and response to biological challenge

**Figure 6:**
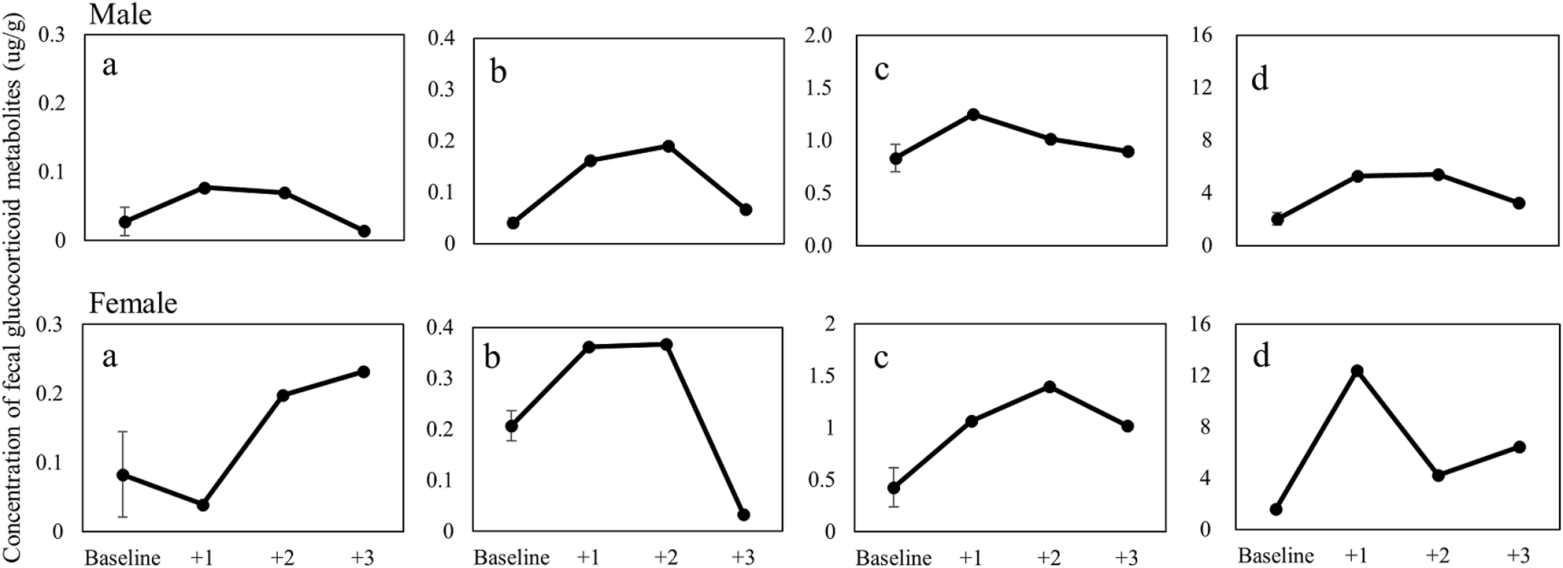
Concentrations of FGMs before and after anesthesia stimulation of one male and one female Formosan macaque measured using four EIA assays. The baseline values were average readings of feces collected before anesthesia with standard error. (**a**) Cortisol; (**b**) corticosterone; (**c**) 11-oxoetiocholanolone; and (**d**) 11β-hydroxyetiocholanolone.

The FGM concentrations in feces collected on the days before health checks in all individuals exhibited low variation at baseline, except for cortisol, and a substantial increase after health checks (Fig. 6), ranging from 1.8- to 6.9-fold above the baseline (Table 3). The peaks of each FGM appeared on the first to third days after the anesthesia procedure in all individuals and gradually returned to baseline.

However, only 11β-hydroxyetiocholanolone and 11-oxoetiocholanolone showed measurable peaks in the HPLC profiles. Furthermore, coelution with androgen metabolites was not observed in HPLC fractions in all EIAs (Fig. 6). In addition, the baseline values of all tested individuals showed low variations, except cortisol. On the basis of the four criteria for the FGM EIA assay validation proposed by Heistermann et al.^30^, we determined that cortisol and corticosterone were unsuitable EIAs for FGM analysis in Formosan macaque. The response of 11-oxoetiocholanolone after anesthesia stimulation was only 1.8-fold more than the baseline value in the male macaque. Therefore, we considered that 11β-hydroxyetiocholanolone was suitable for FGM analysis for monitoring adrenocortical activity in both sexes of Formosan macaques. However, 11-oxoetiocholanolone was suitable for use only for the female macaque (Table 3).

**Table 3:** Increase in FGMs (in folds) compared with baseline measured using four EIAs in response to anesthesia stimulation in both sexes.

EIAs	Male	Female
Cortisol	2.8	2.8
Corticosterone	4.6	1.8
11-oxoetiocholanolone	1.8	**3.0**
11β-hydroxyetiocholanolone	**2.8**	**6.9**

Numbers in bold indicate that the EIA was considered suitable for monitoring adrenocortical activity in a specific sex of Formosan macaques. The suitability evaluation was based on the study by Heistermann et al.^30^.

### Analysis of FGMs from Formosan macaques with and without alopecia by using EIAs

**Figure 7:**
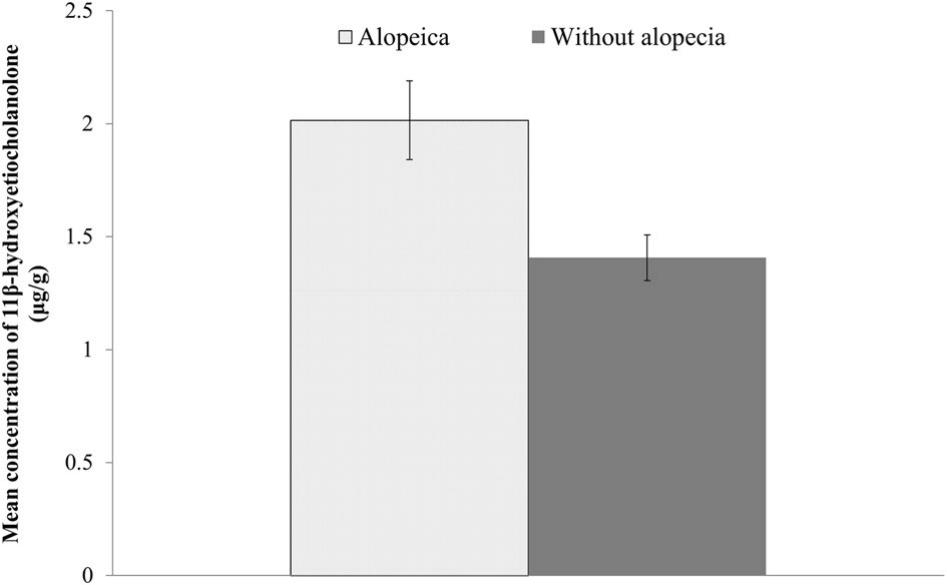
Mean concentration and standard error of 11β-hydroxyetiocholanolone of fecal samples collected from Formosan macaques with and without alopecia in Mt. Longevity.

We collected fresh fecal samples of identified individuals by following the movement of troops from August 2015 to August 2016. In total, 41 fecal samples were collected from 10 individuals with alopecia and 31 individuals without alopecia (Table 4; Table S1). We selected the EIAs of 11-oxoaetiocholanolone and 11β-hydroxyetiocholanolone for FGM assay in Formosan macaques based on EIA validation assays. Mean concentrations (standard error) of 11-oxoaetiocholanolone in individuals with and without alopecia were 1.47 (0.16) and 1.12 (0.07) μg/g, respectively (Table 3). Furthermore, mean concentrations (standard error) of 11β-hydroxyetiocholanolone in individuals with and without alopecia were 2.02 (0.17) and 1.41 (0.10) μg/g, respectively (Table 4, Fig. 7).

**Table 4:** Individual characteristics of fecal samples of Formosan macaques with and without alopecia in Mt. Longevity and the results of FGM concentration through EIA analysis.

Alopecia	Female			Male			Mean concentration of FGMs (μg/g^a^)
	Adult	Juvenile	Subadult	Adult	Juvenile	Subadult	11β-hydroxy (SE)^b^
No	12	2	1	11	2	3	1.41 (0.10)
Yes	5	1	0	3	1	0	2.02 (0.17)
Total	17	3	1	14	3	3	1.56

^a^Dry weight of fecal sample.

^b^Mean and standard error of 11β-hydroxyetiocholanolone.

We adopted a logistic regression model to evaluate the association between alopecia and explanatory variables, including age, sex, concentrations of 11β-hydroxyetiocholanolone. No variable was discard from the logistic regression model based on the VIF value. Results indicated that the FGM concentrations of 11β-hydroxyetiocholanolone (*p* = 0.012) were significantly positively associated with alopecia when the variable was analyzed alone (Table 5). Age and sex were not associated with alopecia (Table 5). In addition, we evaluated the full model, including the explanatory variables of 11β-hydroxyetiocholanolone, age, and sex, fitted in the logistic regression. In the full model, 11β-hydroxyetiocholanolone was the only variable that showed a significant positive association with alopecia. The AIC value of the full model was 40.49, which was not different from the model with 11β-hydroxyetiocholanolone fitted alone. The results indicated that 11β-hydroxyetiocholanolone better explained the macaque alopecia in our study.

**Table 5:** Explanatory variable statistics and AIC values of each variable tested alone and the full model in the logistic regression analysis by using Formosan macaque with and without symmetrical alopecia as the dependent variable.

Variables	Coefficient	SE	p value	AIC^a^
**Univariate analysis**				
Null model				47.55
FGM concentration				
11β-hydro^b^	1.888	0.755	0.012*	41.53
Sex				
Female	(Reference)			
Male	15.96	2284.1	0.994	48.12
Age				
Juvenile	(Reference)			
Subadult	− 16.573	1978	0.993	47.18
Adult	0.33	0.891	0.711	49.41
**Multivariable analysis**				
FGM concentration				
11β-hydro	2.397	0.922	0.009*	40.49
Sex				
Female	(Reference)			
Male	19.629	54,664	0.997	
Age				
Juvenile	(Reference)			
Subadult	− 21.0	4106.1	0.996	
Adult	− 2.067	1.851	0.264	

*p < 0.05.

^a^Akaike information criterion.

^b^11β-hydroxyetiocholanolone.

## Discussion

This study was conducted to identify the possible etiology of symmetrical alopecia in free-ranging Formosan macaques in Taiwan. To our knowledge, bilateral symmetrical alopecia has never been described in a free-ranging macaque species. Moreover, although bilateral symmetrical alopecia has been commonly observed in macaques in captivity, the etiology is still largly unclear^14, 52^. For identifying the etiology, we first evaluated the histopathological changes in skin tissues with symmetrical alopecia and observed that the histopathological characteristics of alopecia were similar to those of telogen effluvium^53–55^. The environmental stressor that induced telogen effluvium was suspected based on the observation of individuals with symmetrical alopecia. For assessing the relationship between symmetrical alopecia and stress, we validated the EIA of 11β-hydroxyetiocholanolone for Formosan macaque FGMs using noninvasive fecal samples. In addition, we found that the concentration of 11β-hydroxyetiocholanolone was positively associated with symmetrical alopecia.

Physiological or emotional stress is commonly identified as a cause of telogen effluvium in humans^56^. Other potential triggers identified in humans include febrile illness, severe trauma, postpartum hormonal changes, malnutrition, hypothyroidism, and iron deficiency^57^. Studies in mice have revealed that stress can induce profound hair growth inhibitory and hair-damaging proinflammatory effects, which generate a telogen effluvium pattern^15, 58^. Telogen effluvium alopecia have been described in squirrel monkey and rhesus macaques^54, 59^. However, both studies have concluded that stress was not the potential trigger based on the dominance hierarchy and blood cortisol concentration^54, 59^. The assessment methods adopted in their studies might be incapable of determining the true status of the stress level in individuals because environmental stressors and restriction-induced stress was not controlled.

The association between stress and alopecia has been demonstrated in different macaque species. A histopathological evaluation has rarely been conducted to identify the alopecia pattern^2, 14, 60, 61^. However, many factors can contribute to alopecia in macaques^14^. Therefore, a skin biopsy of the alopecia site has been commonly recommended for histopathological examination and identification of the potential factor that triggers alopecia^14, 53^. In this study, febrile illness, severe trauma, postpartum hormonal changes, malnutrition, and hypothyroidism were ruled out as potential causes of telogen effluvium based on the field observation and serum chemical analysis of three trapped individuals (Table S2). Stress was considered to be the primary factor inducing telogen effluvium in this study. Moreover, the logistic regression analysis showed that stress was associated with symmetrical alopecia in Formosan macaques in Mt. Longevity. The concentrations of 11β-hydroxyetiocholanolone were associated with symmetrical alopecia in the logistic regression analysis when each variable was tested alone. Moreover, 11β-hydroxyetiocholanolone alone was the best fit model compared with other models, 11β-hydroxyetiocholanolone was the most important explanatory variable and it implies that stress may be one of the primary factors of alopecia in the studied population.

FGM assay validation in a specific species or different sexes of species before adopting the assay for measuring the FGM concentration was recommended and commonly followed as the standard procedure^62^. The validation of FGM analysis is described in different macaque species^30, 63–65^. However, many studies have analyzed macaque FGMs by using assays without appropriate analytical and physiological (or biological) validation^62^. Analytical validation measures the precision, accuracy, sensitivity, and specificity, which might be influenced by the cross-reaction of antibody to other substances^24^. Physiological validation is conducted through the pharmacological stimulation of hypothalamic–pituitary–adrenal (HPA) axis activity. The stimulation affects the amount of GC secretion into the blood, and then noninvasive samples (urine, saliva, or feces) that reflect the predicted changes of GCs in the blood are measured and validated^66^. The Adrenocorticotropic hormone (ACTH) challenge and dexamethasone suppression tests have been commonly adopted for physiological validation^62, 66^. However, these methods have disadvantages, particularly in wild animals, such as unknown dosage for inducing the HPA axis activity and special permission required to conduct animal experimentation^62^. In this case, biological validation, which involves exposing animals to stressors such as restraint, anesthesia, or transportation, is recommended^20, 23, 24, 66^. A proper FGM assay has never been validated and established for Formosan macaques. We evaluated the suitability of four EIAs for FGM assay in this study and determined that the 11β-hydroxyetiocholanolone EIA was suitable for Formosan macaques based on analytical and biological validation. In addition to Formosan macaque, the 11β-hydroxyetiocholanolone EIA is a validated FGM assay in various macaque species^30, 63^. The validated 11β-hydroxyetiocholanolone EIA could be applied for the stress assessment of Formosan macaque in wild and captivity.

In this study, we identified that stress was the most likely factor to trigger symmetrical alopecia in a wild Formosan macaque population in Mt. Longevity. The high density of Formosan macaque population in Mt. Longevity was suspected to be a stressor; however, it remained unclarified. Although stress can decrease the fitness of an individual, considering the population status of Formosan macaques in Taiwan is stable^67^ and alopecia was only observed in our study area, which is isolated from other populations, the impact on the total population of Formosan macaque in Taiwan is limited. Nonetheless, stress-induced immunosuppression and alopecia might affect the local abundance and increase zoonosis risk in Mt. Longevity, where human–macaque contact is frequent^68^. Future studies are suggested to focus on the causative factor of stress and the effects of stress and alopecia on the health and welfare in the Formosan macaques.

## Supplementary Information

Supplementary Table S1.

Supplementary Table S2.
