# Supplementary material for: Association between stress and bilateral symmetrical alopecia in free-ranging Formosan macaques in Mt. Longevity, Taiwan
Source: Sci Rep. 2021 May 27;11:11189. doi: 10.1038/s41598-021-90725-2 (PMC8160012; doi:10.1038/s41598-021-90725-2)
Supplement: Supplementary file 1 — Supplementary Table S1. [file 41598_2021_90725_MOESM1_ESM.docx]

Table S1. Individual characteristics of fecal samples collected from Formosan macaques with or without alopecia in the Mt. Longevity and the result of FGMs concentration using EIAs analysis

| **Sample code** | **Age** | **Sex** | **Alopecia** | **11-oxo^1^** | **11β-hydroxy^2^** |
| --- | --- | --- | --- | --- | --- |
| FF01 | Subadult | Male | No | 1.54 | 1.64 |
| FF02 | Subadult | Male | No | 0.39 | 0.85 |
| FF03 | Adult | Female | No | 0.89 | 1.34 |
| FF04 | Adult | Male | No | 0.74 | 1.37 |
| FF05 | Adult | Female | No | 1.16 | 2.68 |
| FF06 | Subadult | Female | No | 1.3 | 2.38 |
| FF07 | Adult | Male | No | 1.47 | 2.24 |
| FF08 | Adult | Female | Yes | 2 | 2.67 |
| FF09 | Juvenile | Male | No | 1.79 | 1.04 |
| FF10 | Adult | Male | No | 1 | 1.87 |
| FF11 | Adult | Female | No | 0.71 | 1.01 |
| FF12 | Adult | Female | No | 0.75 | 1.02 |
| FF13 | Adult | Female | No | 1.33 | 0.72 |
| FF14 | Adult | Male | No | 0.79 | 2.18 |
| FF15 | Adult | Male | No | 0.65 | 1.52 |
| FF16 | Adult | Male | No | 0.97 | 1.21 |
| FF17 | Adult | Male | No | 0.78 | 1.19 |
| FF18 | Adult | Male | No | 2.17 | 1.66 |
| FF19 | Adult | Male | No | 1.28 | 2.23 |
| FF20 | Adult | Female | No | 0.97 | 1.42 |
| FF21 | Adult | Female | No | 0.98 | 1.42 |
| FF22 | Juvenile | Male | No | 1.02 | 2.19 |
| FF23 | Adult | Male | Yes | 1.09 | 2.07 |
| FF24 | Adult | Male | Yes | 0.83 | 1.94 |
| FF25 | Adult | Male | Yes | 1.73 | 2.86 |
| FF26 | Adult | Female | No | 1.64 | 0.67 |
| FF27 | Adult | Female | No | 1.19 | 1.56 |
| FF28 | Juvenile | Female | Yes | 1.62 | 2.12 |
| FF29 | Adult | Female | No | 1.32 | 0.57 |
| FF30 | Subadult | Male | No | 0.7 | 0.56 |
| FF31 | Adult | Female | Yes | 0.56 | 0.91 |
| FF32 | Adult | Female | Yes | 1.44 | 1.99 |
| FF33 | Adult | Female | No | 1.72 | 0.78 |
| FF34 | Juvenile | Male | Yes | 1.88 | 1.81 |
| FF35 | Adult | Female | Yes | 1.92 | 1.52 |
| FF36 | Adult | Male | No | 1.71 | 1.43 |
| FF37 | Juvenile | Female | No | 1.24 | 1.13 |
| FF38 | Adult | Female | Yes | 1.67 | 2.26 |
| FF39 | Adult | Male | No | 0.93 | 1.49 |
| FF40 | Juvenile | Female | No | 0.61 | 0.96 |
| FF41 | Adult | Female | No | 1.06 | 1.28 |

^1^ 11-oxoaetiocholanolone (ug/g)

^2^ 11β-hydroxyaetiocholanolone (ug/g)
