# Supplementary material for: Association between stress and bilateral symmetrical alopecia in free-ranging Formosan macaques in Mt. Longevity, Taiwan
Source: Sci Rep. 2021 May 27;11:11189. doi: 10.1038/s41598-021-90725-2 (PMC8160012; doi:10.1038/s41598-021-90725-2)
Supplement: Supplementary file 2 — Supplementary Table S2. [file 41598_2021_90725_MOESM2_ESM.docx]

**Supplement Table 2. Serum chemistry analysis of free thyroxine, albumin, vitamin D, and zinc in three trapped alopecia individuals. Hypothryoidism, malnutrition, and low serum concentration of vitamin D and Zinc have been indicated to be related to alopecia in human and different animal species ^1,2^.**

| Individuals | Free T4 (ng/dl) ^a^ | ALB (g/dl)^b^ | 25-OH-D (ng/ml)^c^ | Zinc (µg/dL) |
| --- | --- | --- | --- | --- |
| 2016080401 | 0.885 | 5.1 | 68 | 90 |
| 2016080402 | 0.777 | 6 | 65 | 50 |
| 2016080403 | 1.49 | 5.1 | 70 | 76 |
| Normal value | 1.45±1.23 (RM)^3^ | 4.56±0.23 (CM)^4^  4.5±0.33 (male RM)^5^  4.3±0.58 (female-RM)^5^ | 61±4.0(TM)^6^ | 71±10(RM)^2^ |

^a^ free thyroxine; ^b^ albumin; ^c^25 hydroxyvitamin D, the primary circulating vitamin D metabolite.

Due to the lacking of the normal range of each serum chemistry value for Formosan macaques, we used the values determined from different macaque species to instead. RM denotes Rhesus Macaques; CM denotes cynomolgus monkeys; TM denotes Toque macaque.

**Reference.**

1 Novak, M. A. & Meyer, J. S. Alopecia: possible causes and treatments, particularly in captive nonhuman primates. *Comp. Med.* **59**, 18-26 (2009).

2 Swenerton, H. & Hurley, L. S. Zinc deficiency in rhesus and bonnet monkeys, including effects on reproduction. *The Journal of Nutrition* **110**, 575-583 (1980).

3 Luchins, K. R. *et al.* Application of the diagnostic evaluation for alopecia in traditional veterinary species to laboratory rhesus macaques (*Macaca mulatta*). *J Am Assoc Lab Anim Sci* **50**, 926-938 (2011).

4 Park, H.-K. *et al.* Reference values of clinical pathology parameters in cynomolgus monkeys (*Macaca fascicularis*) used in preclinical studies. *Lab Anim Res* **32**, 79-86, doi:10.5625/lar.2016.32.2.79 (2016).

5 Koo, B.-S. *et al.* Reference values of hematological and biochemical parameters in young-adult cynomolgus monkey (*Macaca fascicularis*) and rhesus monkey (*Macaca mulatta*) anesthetized with ketamine hydrochloride. *Lab Anim Res* **35**, 7, doi:10.1186/s42826-019-0006-0 (2019).

6 Power, M. L. & Dittus, W. P. J. Vitamin D status in wild toque macaques (Macaca sinica) in Sri Lanka. *American journal of primatology* **79**, e22655 (2017).
